# Animal Models of Diabetes Mellitus for Islet Transplantation

**DOI:** 10.1155/2012/256707

**Published:** 2012-12-30

**Authors:** Naoaki Sakata, Gumpei Yoshimatsu, Haruyuki Tsuchiya, Shinichi Egawa, Michiaki Unno

**Affiliations:** ^1^Division of Hepato-Biliary-Pancreatic Surgery, Department of Surgery, Tohoku University Graduate School of Medicine, 1-1 Seiryo-machi, Aoba-ku, Sendai, Miyagi 980-8574, Japan; ^2^Division of International Cooperation for Disaster Medicine, International Research Institute of Disaster Science, Tohoku University, Sendai, Miyagi 980-8574, Japan

## Abstract

Due to current improvements in techniques for islet isolation and transplantation and protocols for immunosuppressants, islet transplantation has become an effective treatment for severe diabetes patients. Many diabetic animal models have contributed to such improvements. In this paper, we focus on 3 types of models with different mechanisms for inducing diabetes mellitus (DM): models induced by drugs including streptozotocin (STZ), pancreatomized models, and spontaneous models due to autoimmunity. STZ-induced diabetes is one of the most commonly used experimental diabetic models and is employed using many specimens including rodents, pigs or monkeys. The management of STZ models is well established for islet studies. Pancreatomized models reveal different aspects compared to STZ-induced models in terms of loss of function in the increase and decrease of blood glucose and therefore are useful for evaluating the condition in total pancreatomized patients. Spontaneous models are useful for preclinical studies including the assessment of immunosuppressants because such models involve the same mechanisms as type 1 DM in the clinical setting. In conclusion, islet researchers should select suitable diabetic animal models according to the aim of the study.

## 1. Introduction

Islet transplantation is a cell replacement therapy for severe diabetes mellitus (DM), including type 1 DM, that has a long history. In 1967, Lacy and Kostianovsky established a method for isolating islets from rat pancreas using collagenase [1]. Their group also first succeeded in islet intraportal transplantation and reversed diabetes in a rat model [2]. In the clinical setting, the first successful trial of human islet allotransplantation was performed at the University of Pittsburgh in 1990, and five patients achieved an insulin-free condition after islet transplantation [3]. Shapiro and colleagues could significantly improve the outcome of islet transplantation using the “Edmonton Protocol” based on multiple transplantations with a steroid-free immunosuppressive regimen in 2000 [4]. Recent data of clinical islet transplantation revealed that 50% of the patients achieved insulin independence for 5 years [5]. This represents great progress since the rate of being insulin-free was only 10% in the late 1990s [6]. Many studies have been done to improve the success of islet transplantation and will continue to be done in the future.

Animal models of DM have contributed much to islet research and to evaluations of isolated human islets for clinical islet transplantation. Good control of blood glucose is the major goal in islet research. Assessments of drugs for improving islet function and immunosuppressants for protecting transplanted islets from the immune system are good examples of progress toward this goal. We introduce some animal models of DM in this paper, and clarify why and how the models should be used, based on our experience and publications by others.

## 2. Animal Models of DM for Islet Research

### 2.1. History of Animal Models of DM

Animal experiments have a long history in the field of diabetic research, including islet research. Animal models of DM are classified as spontaneous or secondary (drug-induced, pancreatectomized, and biomolecular method). The earliest animal model of DM was a pancreatomized dog that was used to evaluate the intestine function in the 1880s, and many pancreatomized animal models (rabbit and dog) have been used for diabetic studies including the purification of insulin [7].

While the pancreatomized model was established approximately 100 years ago, drug-induced models also have a long history. Alloxan is a pyrimidine derivative that was synthesized by Wöhler in 1838 [8], and it was considered a representative drug for diabetes by inducing necrosis of the endocrine beta cells in 1940s [9, 10]. The first report of streptozotocin (STZ: N-nitroso derivative of glucosamine), a most convenient drug for inducing diabetes, was published in 1963 [11]. STZ was used as chemotherapeutic agent for cancer based on its inhibition of DNA synthesis and, like alloxan, has been used for making animal models of DM [12].

Spontaneous diabetic animal models were first described in the 1970s. A nonobese diabetic (NOD) mouse, known as a type 1 DM model, was established by Makino's group from female mice derived from a JcI-ICR strain that developed cataracts [13]. The biobreeding (BB) rat, which was established by the Bio Breeding Laboratory and described in 1974 [14], is also an animal model of type 1 DM. Islets of these models suffered from autoimmune attack by T cells, B cells, macrophages, and natural killer cells, inducing insulitis and finally leading to islet loss [15–19]. This mechanism reflects the onset of type 1 DM, and these models have been utilized for many studies about this disease. Drug-induced diabetic models are similar to spontaneous type 1 DM models in terms of the loss of islets and deficiency of internal insulin, but the mechanism is different: the involvement of autoimmunity is a feature of the latter type.

On the other hand, there are many animal models of type 2 DM including *ob/ob* mice in 1949 [20], *db/db* mice in 1974 [21], *fa/fa* (Zunkar) rats in 1989 [22], Goto-Kakizaki rats in 1976 [23], and Otsuka Long-Evans Tokushima fatty (OLETF) rats in 1992 [24]. Many of them are animal models of obesity, and they contribute to clarifying the correlationship between obesity and type 2 DM. *ob/ob* mice and *fa/fa* rats have hyperinsulinemia with insulin resistance when they gain weight [7], reflecting the condition of obesity in human. Recently, it was clarified that a deficiency of or resistance to leptin, a 16 kDa protein hormone that plays a key role in regulating energy intake and energy expenditure, caused the development of obesity in *ob/ob* mice (this is a model of ob gene deficient) [25], *fa/fa* rats, and *db/db* mice (which are leptin-resistant models due to a mutation of the leptin receptor) [26, 27].

Transgenic animal models have been used in diabetic research since the 1990s, and such models have revealed the roles of many genes that are related to DM including type 1 and 2 DM. Insulin receptor gene knockout mice have severe hyperglycemia and neonatal death by ketoacidosis [28]. Moreover, insulin receptor substrate (IRS), which is known to be an important ligand in the insulin response, has a different function according to the type of animal. For example, IRS1 gene knockout mice have mild insulin resistance with normoglycemia [29], while IRS2 gene knockout mice suffer from severe hyperglycemia with insulin resistance and reduction of the *β* cell mass [30, 31]. These findings make understandable the fact that some type 2 DM patients have normoglycemia or mild hyperglycemia, while others have severe hyperglycemia.

Many animal models of DM have been developed and are chosen depending on the purpose of the study (Table 1). In islet transplantation research, STZ-induced diabetic animals are mainly used, while spontaneous and pancreatomized models are also available.

### 2.2. STZ-Induced Diabetic Model for Islet Transplantation

#### 2.2.1. Background

STZ, a white powder with a molecular weight of 265.221 g/moL, is a glucosamine-nitrosourea compound. STZ causes toxicity to cells by impairing DNA and by other mechanisms. One is the activation of poly-ADP ribosylation, which is likely more important for the induction of DM than the DNA damage [32]. STZ is similar to glucose insofar as it is transported into *β* cells by the glucose transport protein GLUT2, but is not recognized by the other glucose transporters. Thus, *β* cells have relatively high levels of GLUT2 [33, 34]. This explains the specific toxicity of STZ for *β* cells. In the early 1970s, Lacy and his collaborators first performed experimental islet transplantation to rats with STZ-induced DM [35, 36]. STZ was applied not only to rats but also many other kinds of mammals including mouse [37], monkey [38], and dog [39]. Since then, STZ-induced diabetic models have been available for islet research.

#### 2.2.2. Dose and Method of Inducing DM

The dose of STZ is varied according to the animal species and strain (Table 2) [40–87]. For example, mice required higher doses of STZ for inducing DM compared with other animals. While in ICR mice DM can be induced with comparatively lower doses (90–150 mg/kg), C57BL/6 mice need approximately 200 mg/kg dose of STZ [40, 42–48]. Immunodeficient mice tend to need less dose of STZ than other wild-type strains. For example, NOD-SCID mice are induced DM with 120–140 mg/kg dose of STZ [88–90]. STZ injection is done with two methods: a single injection and low dose multiple injection. Continuous low dose injection has been used in many studies, especially these employing rodents [49–57]. The merit of single injection is its convenience (only 1 injection), but the diabetic model induced by this method is unstable in terms of the diabetic condition, and sudden death is caused in approximately 10%–20% of the animals due to the elevation of blood glucose and the toxicity of STZ. On the other hand, the low dose multiple injection method can achieve hyperglycemia with no or few dead cases [91]. It is considered that the low dose multiple injection model is superior to single injection model in terms of safety. Intraperitoneal (i.p.) injection tends to be employed for rodents while intravenous (i.v.) injection used for larger animals, because i.p. is easier than i.v. in rodents while i.v. has the merit of direct injection of STZ without loss of STZ in comparison with i.p. Therefore i.v. injection can induce diabetes at a lower dose of i.p. because more time is required to absorb STZ with i.p., and some of the STZ is inactivated before absorption. The dose of STZ for i.v. is approximately 90% of that for i.p. in previous publications (Table 1). Citrate buffer at pH 4.5 has been used as a solvent in many studies because STZ remains stable at low pH (Table 2), but there is no problem with using normal saline if STZ is used immediately after dissolving.

#### 2.2.3. Our Method of DM Induction Using STZ and Discussion of STZ-Induced DM Model for Islet Transplantation

**Figure 1:**
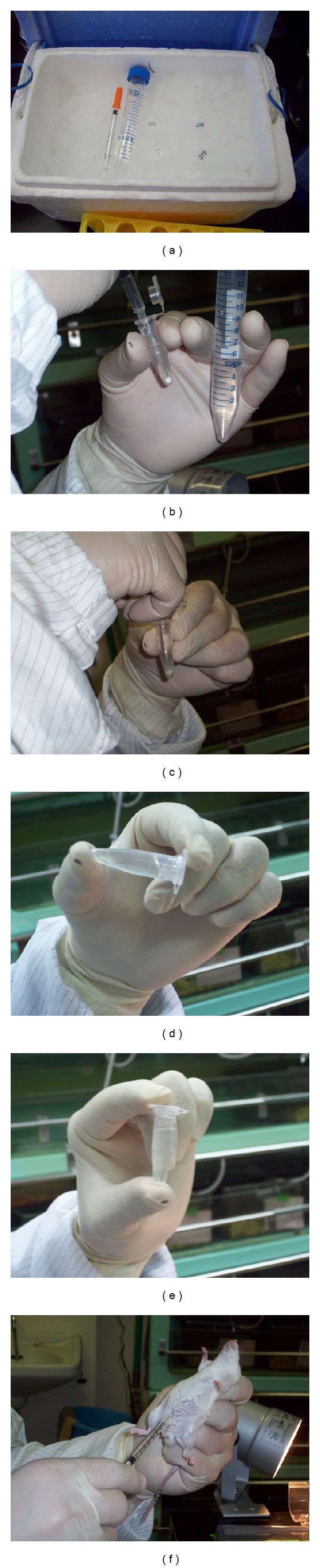
STZ injection via intraperitoneum. (a) Preparation. 1.5 mL centrifuge tube with STZ, 29-gauge needle, 1 mL syringe, ice box with crushed ice, citrate buffer solution, and an 18-gauge needle are used. (b)–(e) Dissolving STZ. (b) Citrate buffer solution is poured into STZ. (c) Crushing the larger clusters of STZ with an 18-gauge needle. (d) and (e) Dissolving by shaking gently. (f) STZ solution is injected via the lower abdomen with care not to injure organs.

**Figure 2:**
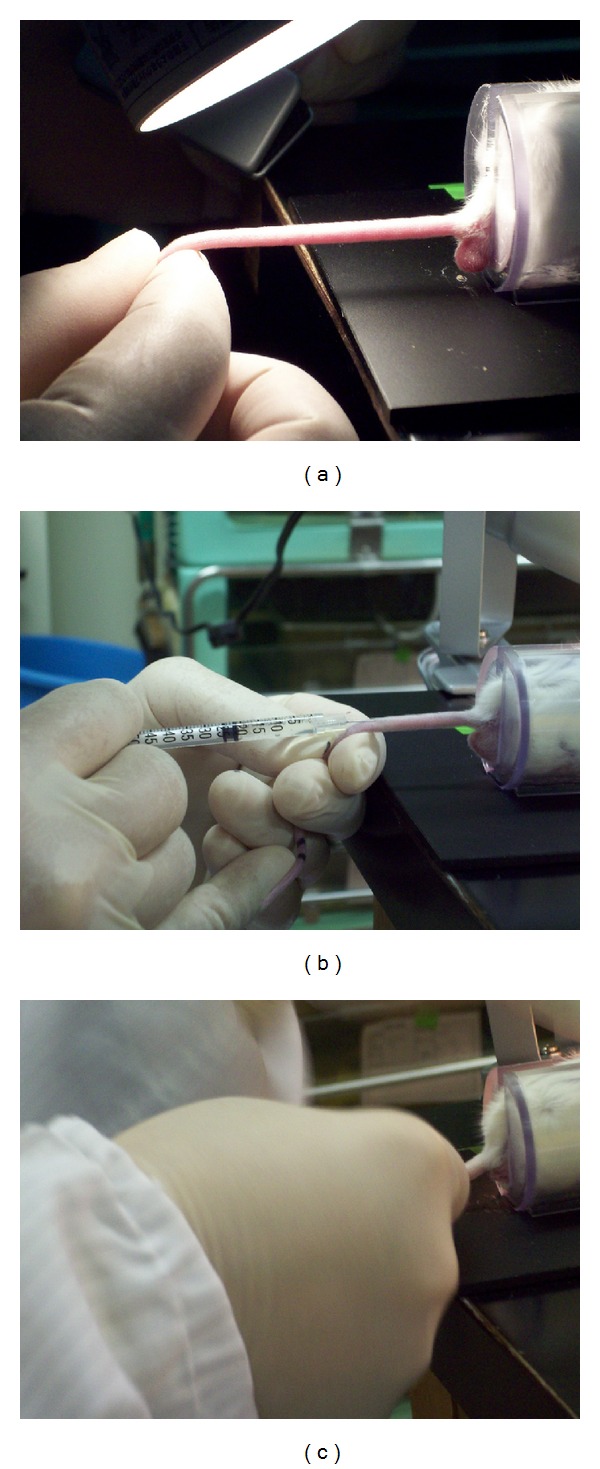
STZ intravenous injection. (a) Dilating tail vein by warming (using light in this case). (b) Injecting solution via the tail vein. A 29-gauge needle is inserted at the upper 1/2~1/3 of the tail. STZ solution is injected slowly. (c) After finishing injection, the needle is removed while pressing a part of puncture with the thumb to prevent leakage of the STZ solution.

**Figure 3:**
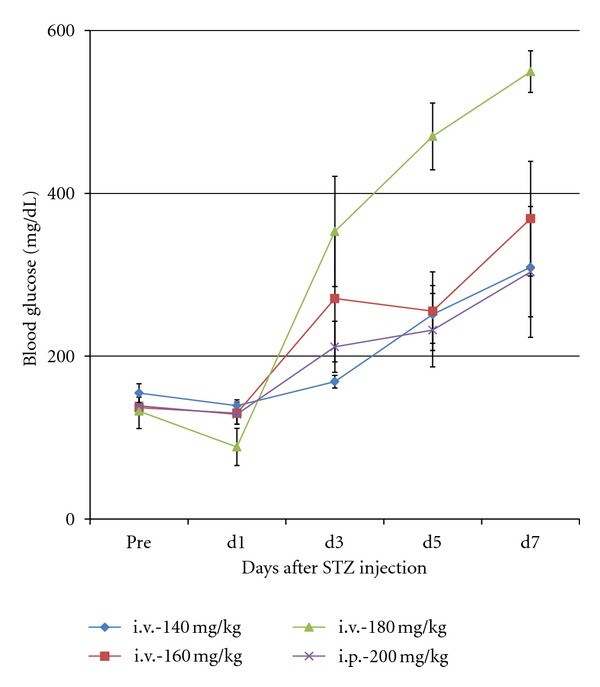
Blood glucose levels after STZ injection in BALB/c mice. The effect of 160 mg/kg of STZ injection via i.v. was equally to that of 200 mg/kg of STZ injection via i.p. in blood glucose level.

STZ (Sigma-Aldrich Co. LLC., St. Louis, MO, USA) must be stored in a freezer (under −20°C condition) because of its instability. To prevent inactivation, we divide 20 mg of STZ into 1.5 mL centrifuge tubes before using. Some of the divided STZ (3-4 mice per tube), 1 mL syringe with 29-gauge needle, an ice box with crushed ice, the solvent (citrate buffer solution, 0.09 M, pH4.8 (Sigma-Aldrich) or normal saline), and 18-gauge needles are prepared for the treatment (Figure 1(a)). The STZ tubes should be placed on ice until dissolution. At first, the mice are marked on the tail and measured for body weight, and then the final volume of STZ solution is decided. We induce DM with STZ at 200 mg/kg body weight. 20 mg of STZ is dissolved with 1 mL of solvent (the concentration is 20 mg/mL). We crushed the larger clusters of STZ with a 18-gauge needle and then dissolved it by shaking gently (Figures 1(b)–1(e)). To induce DM for a mouse with 20 g of body weight, for example, 200 *μ*L of STZ solution is necessary. STZ solution should be used as soon as possible after dissolving, because the diabetogenic activity of STZ is decreased in the solvent [92]. Injection should be done within 10 minutes after the dissolution. In the case of i.p., the STZ solution is injected via the lower abdomen with care taken to prevent organ injury (Figure 1(f)). In the case of i.v., STZ solution is injected via a tail vein. Tail veins are located at the dorsal and left and right sides of the tail. After dilating the tail vein by warming, the 29-gauge needle is inserted in the upper 1/2~1/3 of the tail and the STZ solution is injected slowly (Figures 2(a) and 2(b)). After finishing the injection, the needle is removed while pressing part of the puncture with thumb to prevent leakage of the STZ solution (Figure 2(c)). After the injection of STZ, we check blood glucose every day, and mice with over 350 mg/dL of blood glucose at two consecutive measurements are considered DM mice suitable for islet transplantation. Our data revealed that the effect of 160 mg/kg of STZ injection via i.v. was equal to that of 200 mg/kg of STZ injection via i.p. in blood glucose level (Figure 3). The rates of achieving hyperglycemia (over 350 mg/dL) in BALB/c mice were 58.8% (33.3%–70%, *n* = 36) by i.p. method and 81.8% (80%–83.3%, *n* = 22) by i.v. method. In our opinion, diabetic models for islet transplantation studies should have severe hyperglycemia because mice with mild hyperglycemia (under 300 mg/dL) can show normoglycemia depending on the measurement time, and it can be difficult to determine whether the normoglycemia is due to the original pancreatic endocrine function of the recipient mice or due to the islet transplantation. Fasting is not necessary, but the measurements for blood glucose should be done at the same time to prevent the influence of ingestion. We measure the blood glucose from 6 to 10 AM. The afternoon is excluded because the blood glucose in the afternoon tends to be higher than in the morning. We defined normoglycemia as under 200 mg/dL blood glucose because it is the upper range of blood glucose in normal (untreated) mice.

### 2.3. Pancreatomized Diabetic Model for Islet Transplantation

Pancreatomized animals are one of the oldest models for DM. This model was considered to be and used as a type 1 DM model because of the elimination of the pancreatic *β* cells. The difference between this model and type 1 DM is the stability of the blood glucose: the type 1 DM model has a deficiency of insulin release but preservation of other hormonal functions that act to increase the blood glucose (i.e., glucagon). Pancreatomized diabetic animals have been used as islet autotransplantation models. Minnesota group performed pancreatectomy and islet autotransplantation in pigs in 1976 [93]. This group also performed islet autotransplantation of pancreatomized dogs and succeeded in achieving normoglycemia at the same time [39]. After their success, many experimental models of total pancreatectomy with islet autotransplantation using dogs were published in the 1970s and proved the effectiveness of the treatment [94–96]. These findings contributed to clinical trials of total pancreatectomy with islet autotransplantation for chronic pancreatitis [97]. Large pancreatomized animal models have also been used for the evaluation of cryopreserved islets [98–101] and optimal transplant sites [102–107]. Large pancreatomized diabetic animal models have been used not only for islet autotransplantation studies but also for allotransplant studies. Many groups examined the usefulness of immunosuppressants including cyclosporine using islet allotransplanted dog [108–110] and pig models [111]. Pancreatomized diabetic large animals were also used for studies about bioartificial pancreas (i.e., islet encapsulation technology using high molecular compound to prevent from immune attack) in allo- and xenotransplantation [112–118]. Large pancreatomized animal models for islet transplantation, including pig, dog, and monkey, are considered preclinical models and tend to be selected for evaluating some treatments in the clinical setting.

One of the recent uses of pancreatomized models is to assess *β* cell regeneration after pancreatectomy. Pancreatectomy over 70% induces some pancreatic regeneration-related factors like pancreatic and duodenal homeobox 1 (PDX1) and neurogenin-3 (Ngn3), which contribute to the regeneration of *β* cells in rodent models [119–121]. Recently, Jung and colleagues revealed that the regeneration of *β* cells could be detected in the remnant pancreas of BALB/c mice with 70% pancreatectomy, and the effect of regeneration was more prominent in pancreatomized mice with islet autotransplantation [122]. It is interesting that islet masses in remnant pancreas were increased with greater expression of Ngn3 and BrdU (a proliferation marker) and lower expression of TUNEL (an apoptosis marker) in pancreatomized mice with islet autotransplantation compared to pancreatomized mice without transplantation. They concluded that insulin released from transplanted islets has a role in the induction of islet proliferation and antiapoptotic effects on remnant islets. Pancreatomized animal is an old diabetic model, but is still important and useful for preclinical trials on islets transplantation and for understanding the mechanism of DM, including islet regeneration.

### 2.4. Spontaneous Type 1 Diabetic Models for Islet Transplantation

Spontaneous type 1 diabetic animals are useful models to understand the mechanism of type 1 DM because this model has autoimmunity, which causes islet injury like that found in type 1 DM patients. In general, it is difficult to study this disease in human and also difficult to analyze the findings in patients who received islet transplantation because of ethical considerations [123]. This is also a reliable model for analyzing the induction of transplantation tolerance [124] and the special problems of tolerance induction by autoimmunity [125]. The represented spontaneous diabetic models are BB rat and NOD mouse.

The mechanism of inducing DM in NOD mice is insulitis, which is inducted at 4 to 5 weeks after birth, and results in approximately 90% of females and 60% of males developing DM when they are 7 months old [123]. The autoimmunity of NOD mice is characterized by many abnormalities in the immune system including defects in regulatory T cells, which also appears in human in type 1 DM [123]. Regulatory T cells have a role of maintaining immune tolerance. Thus, it is considered that a defect in regulatory T cells causes them to be resistant to tolerance in islet transplantation [126]. As a model of type 1 DM, NOD mice have been used for the assessments of many immunosuppressants.

The first successful regime of immunosuppressants for islet transplantation was a combination of an anti-interleukin (IL)-2 monoclonal antibody daclizumab for the induction, the calcineurin inhibitor tacrolimus, and the mammalian target of rapamycin (mTOR) inhibitor sirolimus for the maintenance referred to as the Edmonton protocol in 2000 [4]. The utility of the regime was proved by the Miami group with an animal study using NOD mice [127]. This group performed islet allotransplantation using diabetic NOD mice and confirmed that a combination of tacrolimus and sirolimus could prolong the graft survival (over 100 days in 28.6% of recipient mice) and a combination of the three agents could achieve even better graft survival (over 100 days in 75%). The Minnesota group examined the effectiveness of antithymocyte globulin (ATG) and the antitumor necrosis factor (TNF)-*α* receptor inhibitor etanercept for the induction as a novel regime for single donor transplantation and achieved 62.5% insulin-free in 1 year after transplantation [128]. Recently, they assessed a novel immunosuppressant regime using FcR nonbinding anti-CD3 antibody or T cell-depleting antibodies and a TNF-*α* inhibitor by comparing the recipients enrolled in the Collaborative Islet Transplant Registry and revealed that the combination could result in an insulin-free rate of 5 years (approximately 50%) [129]. These novel immunosuppressants were also evaluated using NOD mice by many groups. Vergani and colleagues compared between treatment of ATG and cytotoxic T-lymphocyte antigen 4 and antibodies (CTLA4-Ig) and no immunosuppressants in the transplantation of BALB/c mice islets to NOD mice and revealed prolonged graft survival [130]. Anti-CD 3 monoclonal antibody has been shown to be effective in suppressing autoimmunity. Baeke and colleagues revealed that a combination of anti-CD3 monoclonal antibody, ciclosporin A, and vitamin D prolonged the graft survival in islet allotransplanted diabetic NOD mice by reducing CD4+ T cells and increasing FOXP3 + CD4+ regulatory T cells [131]. Additionally, Mamchak and colleagues proved that anti-CD3 monoclonal antibody treatment with oral insulin intake improved the blood glucose level of diabetic NOD mice by increasing regulatory T cells and decreasing CD4 + CD25+ T cells, indicating a suppression of autoimmunity [132].

To succeed in clinical islet transplantation, it is necessary to control both allo- and autoimmunity. NOD mice are suitable for evaluating the influences of both types of immunity in islet transplantation.

## 3. Conclusion

In summary, many diabetic animal models have been used for assessing the effects of therapeutic interventions on the outcome of islet transplantation. All the animals that we mentioned were models with a deficiency in islet function, but the mechanisms by which they induce DM are significantly different, and, thus, it is important to select the model according to the aim of the study.

## Figures and Tables

**Table 1:** Animal models of DM.

Characters	Name	Species
Type 1 DM		
Spontaneous model	NOD	Mouse
	BB	Rat

Type 2 DM		
	*ob/ob *	Mouse
	*db/db *	Mouse
Obesity model	*fa/fa *	Rat
	Goto Kakizaki	Rat
	OLETF	Rat

Type 1 and 2 DM		
Drug-induced model	Alloxan	*
	STZ	*

Pancreatic DM		
Pancreatomized model	Pancreatectomy	**

Others		
	Insulin knockout	Mouse
Transgenic animal model	IRS1 knockout	Mouse
	IRS2 knockout	Mouse

*All experimental animals are available.

**All experimental animals are available but larger animals tend to be used.

**Table 2:** Dose of STZ.

Animal species	Animal strains	Dose of STZ (mg/kg body weight)	Time of injection	Solvent	Method of injection	References
	ICR	90–150	1	Citrate buffer, pH 4.5	i.p	[40, 43, 45, 48]
		70	3	Citrate buffer, pH 4.5	i.p	[58]
		130–225	1	Citrate buffer, pH 4.5	i.p	[42, 44, 46, 47]
Mice	C57BL/6	75–100	3		i.v	[59, 60]
		40–65	5	Citrate buffer, pH 4.5	i.p	[49–54]
	BALB/c	150	1	Citrate buffer, pH 4.5	i.p	[61]
		50	5		i.p	[62]

		60–85	1	Citrate buffer, pH 4.5	i.p	[60, 63–65]
	Sprague-Dawley	55–60	1		i.v	[66, 67]
		50	6	Citrate buffer, pH 4.5	i.v	[68]
Rat		50–70	1	Citrate buffer, pH 4.5 or Normal saline	i.p	[69–74]
	Wistar	40	5	Citrate buffer, pH 4.5	i.p	[75]
		50–65	1	Citrate buffer, pH 4.5	i.v	[55–57]

Dog	Beagle	30–50	1		i.v	[76–78]

Velvet Monkey	*Chlorocebus aethiops *	45–55	1	Normal saline	i.v	[79, 80]

Cynomolgus Monkey	*Macaca fascicularis *	68–150	1	Citrate buffer, pH 4.5	i.v	[41, 81, 82]

	Yucatan	125	1		i.v	[83]
	Yorkshire	50	1	Citrate buffer, pH 4.5	i.v	[84]
Pig	Landrace	125	1		i.v	[85]
	Dutch Landrace X Yorkshire X Finnish Landrace	120	1		i.v	[86]
	domestic	90	1	Normal saline	i.v	[87]
